# How to Reduce Test Anxiety and Academic Procrastination Through Inquiry of Cognitive Appraisals: A Pilot Study Investigating the Role of Academic Self-Efficacy

**DOI:** 10.3389/fpsyg.2019.01917

**Published:** 2019-08-20

**Authors:** Ann Krispenz, Cassandra Gort, Leonie Schültke, Oliver Dickhäuser

**Affiliations:** Department of Psychology, University of Mannheim, Mannheim, Germany

**Keywords:** educational psychology, test anxiety, academic self-efficacy, academic procrastination, cognitive appraisals, inquiry-based stress reduction

## Abstract

**Background and Objectives:**

Test anxiety can impair learning motivation and lead to procrastination. Control-value theory of achievement emotions (Pekrun, 2006) assumes test anxiety to be a result of students’ appraisals of the testing situation and its outcomes. Modification of cognitive appraisals such as low self-efficacy beliefs is thus assumed to reduce test anxiety and subsequent procrastination. In the present study, we tested the effects of an inquiry-based stress reduction (IBSR) intervention on students’ academic self-efficacy, their test anxiety, and subsequent procrastination in the final stages of an academic term.

**Design:**

Longitudinal quasi-randomized intervention control trial.

**Methods:**

University students identified worry thoughts regarding a specific and frightening testing situation. Intervention participants (*n* = 40) explored their worry thoughts with the IBSR method. Participants of an active waitlist control group (*n* = 31) received the intervention after the study was completed. Dependent variables were assessed before and after the intervention as well as at the end of the term.

**Results:**

Data-analyses revealed that the IBSR intervention reduced test anxiety as well as subsequent academic procrastination in comparison to the control group. The effect on test anxiety was partly due to an enhancement of self-efficacy.

**Conclusion:**

Our findings provide preliminary evidence that IBSR might help individuals to cope with their test anxiety and procrastination.

## Introduction

*Test anxiety* is a phenomenon well known to many students of different ages. For example, Putwain and Daly (2014) reported 16.4% of English secondary students to suffer from test anxiety. Further, according to Ergene (2003), up to 20% of college students are test anxious. Roughly similar rates were reported by Thomas et al. (2018) who found about 25% of undergraduate university students to be highly test anxious. These prevalence rates are alarming because test anxiety may debilitate academic performance and impair subjective well-being (e.g., Steinmayr et al., 2016). Test anxiety is experienced in achievement contexts that are perceived as potentially threatening to one’s self-esteem (e.g., important exams). Test anxiety is a multidimensional construct (Pekrun, 2006): On a physiological level, test anxious students might experience sweating, palpitations, trembling, and nausea. Cognitively, test anxiety comes along with specific worry thoughts including negative cognitive self-statements regarding academic failure. Additionally, test anxious individuals might experience social worry thoughts as they fear to be negatively judged by teachers, parents, and others (Lowe et al., 2008). On an affective level, test anxiety is associated with unpleasant feelings of agitation, insecurity, and helplessness, which may evoke certain motivational consequences such as avoidance tendencies.

Test anxiety is often accompanied by *academic procrastination* (e.g., Van Eerde, 2003) – the voluntary delay of important and intended actions or decisions against one’s better knowledge and despite the expected negative consequences of the delay and subjective discomfort (e.g., Ferrari et al., 2005). In academic contexts, procrastination occurs for tasks like learning for an exam or writing an essay (Patzelt and Opitz, 2014). It can have serious consequences for students’ academic achievement such as lower grades, longer study periods, as well as premature study drop-out. Helping students to deal with their test anxiety and subsequent procrastination hence seems an effort worth taking. Studies show that *self-efficacy* (Bandura, 1977) – the appraisal of one’s own capabilities to accomplish a specific task (e.g., studying successfully for an exam) – might play an important role in the causation of test anxiety and subsequent procrastination (e.g., Yerdelen et al., 2016). Based on these ideas, the aim of the present paper is to investigate the effectiveness of an intervention in reducing students’ test anxiety and subsequent procrastination by enhancing students’ academic self-efficacy.

### The Relationship Between Test Anxiety and Self-Efficacy

According to control-value theory of achievement emotions (Pekrun, 2006), test anxiety results from an interaction of cognitive control and value appraisals regarding a specific achievement situation (e.g., an upcoming exam). While value appraisals refer to the value students subjectively attribute to achievement activities (e.g., learning for the exam) and their outcomes (e.g., passing the exam), control appraisals refer to students’ assessment of their subjective control regarding these achievement activities and their respective outcomes. In particular, test anxiety is assumed to arise when students focus on a pending achievement situation of high personal value (e.g., a final exam) while only feeling medium in control of their achievement activities. Such anxiety-causing control appraisals can be a consequence of low self-efficacy expectations: As students with low self-efficacy expectations do not believe that they can accomplish a specific learning task (Bandura, 1977), their control expectancy regarding the respective achievement situation is also assumed to be negatively affected. This theoretical notion is supported by empirical studies showing that students with lower self-efficacy expectations also report higher levels of test anxiety (Haycock et al., 1998; Yerdelen et al., 2016).

### The Relationship Between Procrastination and Self-Efficacy

According to temporal motivation theory (TMT; Steel, 2007), procrastination is (just as test anxiety) a function of expectancy and value appraisals regarding the respective learning task and its outcomes. In particular, procrastination is assumed to be more likely for tasks of low value and low expectancy. Further, the expectancy component of procrastination is theoretically predicted to be most strongly influenced by students’ self-efficacy expectancies. In line with these assumptions, empirical studies show that procrastination is more likely for students who do not believe to have the capabilities to study successfully for an exam (Yerdelen et al., 2016).

To sum up, both control-value theory (Pekrun, 2006) as well as TMT (Steel, 2007) assume that low self-efficacy expectancies – amongst other variables – might cause test anxiety and procrastination as they strongly influence students’ perceived control over achievement activities and their outcomes.

### The Causal Relationship Between Test Anxiety and Procrastination

Test anxious students experience increased states of unpleasant physical arousal as well as aggravating worry thoughts (Pekrun, 2006). As a consequence, test anxious students often feel the desire to withdraw from the situation (Geen, 1987; Matthews et al., 1999). Accordingly, meta-analytical studies find a moderate positive association between test anxiety and procrastination (Van Eerde, 2003; Steel, 2007) – students experiencing higher test anxiety also report higher levels of procrastination. However, these results stem from correlational studies, in which test anxiety and procrastination were only measured at single points in time. From a longitudinal perspective (i.e., over the course of an academic term), the causal interplay between test anxiety and procrastination might be more complex (Pekrun et al., 2007). In particular, TMT (Steel, 2007) suggests that procrastination is *not* always a mandatory consequence of test anxiety. In particular, TMT assumes students to procrastinate primarily when deadlines and exams are still far ahead (e.g., at the beginning of an academic term). The validity of this assumption is supported by results of longitudinal studies. For example, Tice and Baumeister (1997) found procrastinators to report lower stress than non-procrastinators, but only in the *early* stages of the academic term. Further, Yerdelen et al. (2016) found a *negative* association between students’ individual trajectories of test anxiety and procrastination throughout 8 weeks of an academic term. While participants’ anxiety significantly decreased over these weeks, their procrastination significantly increased over the same time interval. The authors concluded that the participants might have used procrastination as an emotional coping strategy to help them deal with their initial test anxiety. Unfortunately, the study of Yerdelen et al. (2016) does not provide any information about the causal interplay of test anxiety and procrastination at the *last stages* of the academic term. However, according to TMT, for this time period, students are assumed to procrastinate *less*: As deadlines approach, they are forced to engage in more active coping strategies (such as studying for the exam) if they want to avoid failing due to poor preparation. In line with these assumptions, studies found students to experience higher levels of anxiety (Lay et al., 1989) and stress (Tice and Baumeister, 1997) before exams when they had delayed studying earlier in the semester. Summing up, delaying learning activities (i.e., procrastination) might help students to emotionally cope with their test anxiety in the short run. However, students’ procrastination should decrease at the last stage of an academic term when deadlines and exams are approaching.

### Interventions to Reduce Test Anxiety and Procrastination

There is a wide variety of interventions focusing on test anxiety and/or procrastination. In their review of recent test anxiety interventions, Von der Embse et al. (2013) found that students with high test anxiety can be best supported by multi-method cognitive-behavioral interventions as well as more specific cognitive or behavioral interventions. With regard to procrastination interventions, recent meta-analyses (Rozental et al., 2018; Van Eerde and Klingsiek, 2018) showed that cognitive-behavioral therapy may help students showing high rates of procrastination. From the perspective of control-value theory (Pekrun, 2006) and TMT (Steel, 2007), a cognitive modification of low self-efficacy expectancies seems promising in order to reduce both test anxiety and procrastination. Accordingly, some interventions for test anxiety and procrastination focus on the change of (irrational) beliefs and thought patterns (Pekrun and Stephens, 2009). For example, in rational-emotive behavioral therapy (Ellis, 2002), students are encouraged to question their own thinking patterns with techniques such as direct cognitive debate and logical persuasion in order to replace dysfunctional and irrational beliefs with more realistic ones. However, a permanent modification of cognitive appraisals (such as low self-efficacy expectancies) should not be restricted to rational (i.e., conscious, logical, and reason oriented) debate only. Rather, dual-process models such as cognitive-experiential self-theory (CEST; Epstein, 2003) assume that rational information processing is always – mostly preconsciously and automatically – influenced by implicit schemas learned from past experiences. Thus, cognitive appraisals are never completely based on rational considerations but always biased by experience-based information processing. In accordance with these assumptions of CEST, self-efficacy theory (Bandura, 1977) posits that self-efficacy beliefs stem not only from verbal persuasion, but also from experiential knowledge such as (vicarious) mastery experiences and the current experience of physical arousal. Consequently, the successful modification of cognitive appraisals (such as low self-efficacy beliefs) needs to include rational debate as much as new (self-efficacy enhancing) experiences.

A standardized method that combines an experiential and a rational approach to modify cognitive appraisals is inquiry-based stress reduction (IBSR; Mitchell and Mitchell, 2003). The IBSR method uses a specific set of questions to allow for the identification and exploration of stressful cognitions (e.g., “I am not able to study sufficiently”). In a first step, participants reflect on the emotions (e.g., test anxiety), effects (e.g., procrastination), causes (e.g., negative experiences in school), benefits (e.g., short-term relief from anxiety), and dysfunctionality (e.g., lower achievement) of their stressful cognition in an experiential manner. In a second step, participants are encouraged to imagine reality *without* the distortions caused by the stressful cognition, this way allowing for a new and potentially more positive experience (e.g., feelings of relief or curiosity). In a last step of the IBSR method, participants are guided to find concrete evidence for the validity of the *opposite* of their stressful cognitions (e.g., “I am able to study sufficiently”) and to explore whether the opposite could also be true. This is done in order to help them overcome the tendency to seek or interpret evidence in ways that are biased by already existing beliefs (i.e., the confirmation bias; Nickerson, 1998). This approach can be assumed to be effective as the new-found arguments are self-created and this way more convincing (Briñol et al., 2012). In sum, IBSR should allow for a debate of stressful cognitions through experiential self-exploration *and* rational persuasion.

First empirical evidence points to the potential of IBSR to reduce anxiety. In a single-group study (Leufke et al., 2013), participants of a non-clinical sample received an IBSR intervention. Results revealed that participants’ anxiety (amongst other psychopathological symptoms) declined for at least 3 months after the intervention. Similarly, Smernoff et al. (2015) found participants’ anxiety to decline after an IBSR intervention. However, in both studies a control group and randomization were missing. Thus, it remains unclear if the anxiety-reducing effects were caused by the IBSR intervention or if they were due to other factors. Further, in all the reported studies participants received a 9-day IBSR intervention, making participation very time-consuming. This could be a possible obstacle preventing individuals from attendance. These hindrances were overcome in a study by Krispenz and Dickhäuser (2018), who assessed the effects of a short computer-based IBSR intervention on test anxiety in a sample of university students. Using a short-term longitudinal randomized control trial, the treatment group received a 20-min IBSR intervention in which they investigated one individual worry thought regarding an upcoming exam. Results showed that individuals who had received the IBSR short intervention demonstrated significantly lower thought-related test anxiety than participants from the pooled control groups who had either reflected on their worry thought or were distracted from it. However, the study did not allow to test if the effects hold longer than 2 days. Also, some IBSR participants reported difficulties in applying the IBSR method via computer and without further assistance.

### The Present Research

The present research overcomes the impediments of previous studies. In an experimental control trial with a longitudinal design, for the first time, we investigate the effects of a short IBSR intervention on test anxiety and procrastination over the last part of an academic term in a sample of university students suffering from both phenomena. While all study participants learned to identify their worry thoughts regarding their most frightening exam, intervention participants were additionally taught to use the IBSR method to explore and investigate their worry thoughts. Participants’ test anxiety, procrastination, and self-efficacy were assessed immediately before and after the intervention (i.e., in the middle of the academic term) as well as immediately before exams (i.e., at the end of the academic term).

For participants of the *intervention group* (who did receive an IBSR intervention), we firstly expected an increase in self-efficacy (H_1_) as compared to the control group. This increase in self-efficacy in the intervention group was expected to emerge immediately after the IBSR intervention and to last until the end of the semester for the following reasons: Self-efficacy theory (Bandura, 1977) assumes that a permanent modification of low self-efficacy beliefs may follow from rational debate (i.e., verbal persuasion) as well as from new – self-efficacy-enhancing – experiences. Self-efficacy should thus increase for IBSR participants (but not for control participants) due to the IBSR intervention as IBSR allows for a debate of cognitive appraisals such as low self-efficacy beliefs through experiential self-exploration (e.g., by imagination of the testing situation without the distortions caused by participants’ low self-efficacy beliefs) and rational verbal persuasion (e.g., through exploration of the validity of high self-efficacy beliefs). Second, based on the assumptions of control-value theory (Pekrun, 2006) and TMT (Steel, 2007), the predicted increase in self-efficacy was expected to decrease participants’ test anxiety (H_2_) and – as a consequence – their procrastination (H_3_).

In contrast, for participants of the *control group* (who did not receive any real intervention), we had the following predictions: Regarding the last part of the academic term, we expected an increase in test anxiety and a corresponding decrease in procrastination (H_4_). These predictions were based on the theoretical rationales of TMT (Steel, 2007). According to TMT, students should use more active coping strategies than procrastination at the late stages of an academic term to deal with their test anxiety. This notion is further supported by empirical results which show students to experience higher levels of anxiety before exams when they had delayed studying earlier in the semester (Lay et al., 1989). To sum up, we expected a reduction in procrastination for *both* groups, but through different underlying mechanisms: For the intervention group, the reduced procrastination was assumed to be caused by a *decrease* in test anxiety, while for the control group the reduced procrastination was expected be a consequence of an *increase* in test anxiety.

## Materials and Methods

### Participants

The IBSR intervention seminars were held on the campus of the University of Mannheim (Germany). Therefore, participants were recruited via posters, flyers, lecture announcements, and mass-emails at different German universities either in or close to Mannheim (Germany). The study was explicitly announced as an intervention study for students with test anxiety and/or academic procrastination. In total, 84 students were interested in participating. These individuals were pre-screened via telephone in order to provide them with all the necessary information (e.g., possible intervention dates, basic information about the intervention, participants’ chances of being assigned to the waitlist control group). Ultimately, *N* = 71 students (*M*_*age*_ = 21.85, *SD* = 2.94, range = 18–36 years, 63.1% women) with different study subjects decided to actually participate in the study. Regarding this sample, most participants studied economic sciences (47.6%). Participants’ mean study duration was *M* = 3.76 terms (*SD* = 2.00). Participants indicated to have at least one academic exam at the end of the actual term (*M* = 3.95 exams, *SD* = 1.23).

### Design

The study had a 2 × 3 mixed-factors design with the between-subjects-factor intervention (IBSR vs. an active control group). Measures of self-efficacy, test anxiety, and procrastination were taken pre-intervention (time 1), post-intervention (time 2)^1^, and immediately before exams (time 3, follow-up).

### Procedure

By the time we conducted the study and acquired the data, it was neither compulsory nor customary at the University of Mannheim to seek explicit ethical approval for an experimental study including only participants’ self-reports on test anxiety and procrastination. Nevertheless, we carefully ensured that the study was conducted in line with the ethical guidelines of the American Psychological Association (APA) and in full accordance with the ethical guidelines of the German Association of Psychologists (DGPs): (1) We did not induce test anxiety/procrastination or any other negative states in the participants but merely assessed their thoughts and affect regarding their upcoming exams. We thus had no reasons to assume that our study would induce any negative states in the participants exceeding the normal risks of studying at a university and preparing for exams. (2) The first author is now working at a Swiss university. At this university, she conducted a follow-up study, which explicitly targeted participants with test anxiety and/or procrastination. The human research ethics committee of the respective Swiss university approved this new study. This can be considered as a clear sign that there are no ethical concerns with regard to the procedure of the present study. (3) The study exclusively made use of pseudonymized questionnaires. The data was matched for the analyses using codenames only. Written informed consent was obtained according to the guidelines of the German Psychological Society. Informed consent included information about (a) research object, (b) study and intervention procedure, (c) duration and allowance, (d) possible benefits of participation, (e) anonymity of data collection, and (f) possible risks of participation. Also, participants were explicitly informed that participation was voluntary and could be terminated at any time without any reason or negative consequences for the participant. Participants had to declare that they were at least 18 years old, had read the informed consent, and agreed to the rules of participation.

Participants were pre-screened via telephone interview to ensure they had time to participate on one of the four pre-determined intervention dates. For the first two dates, the control group treatment was scheduled. For the second two dates, the IBSR intervention treatment was scheduled. Participants were assigned to the conditions (IBSR vs. control group) by choosing from the four possible dates without knowing, which treatment was scheduled for the respective dates. Therefore, participants assignment to experimental groups was quasi-randomized. Baseline measures were taken in the middle of the academic term (time 1) and lasted about 45 min. All measures and instructions were paper-pencil based. Assessed were participants’ demographic data as well as study related variables. Then, participants were asked to think of the upcoming academic exams and to consider which of these exams frightened them the most. Next, participants were asked to describe their most frightening exam in detail. Also, participants were asked to rate the exams’ personal value to ensure that participants had actually chosen an exam that was relevant to them. Then, initial levels of self-efficacy, test anxiety, and procrastination regarding the most frightening exam were assessed. One to 2 weeks after baseline-measures were taken, participants of *both groups* attended a first 3-h group seminar held by the first author and another certified IBSR coach. This way, all study participants were given personal attention by the IBSR coaches and participated in social interactions with other participants. In this first 3-h seminar, participants focused on a specific frightening testing situation and in a systematic way wrote down their individual beliefs (e.g., “I am not able to study sufficiently”).

Additionally, participants of the *intervention group* attended another 3-h IBSR seminar (i.e., the actual intervention) and learned to investigate their stressful cognitions with the IBSR method by means of the four questions and several sub-questions (see Table 1). In a first sub-step, the validity of the stressful cognitions was questioned (Questions 1 and 2). Guided by Question 3 and the respective sub-questions, participants reported the mental pictures they associate with the stressful cognitions, their emotions, and bodily sensations. Also, they reflected on the belief’s specific effects, causes, and benefits as well as its functionality. Guided by Question 4, participants were then enabled to perceive reality without the distortions caused by the stressful cognitions and to experience, how they would feel without them. In the third step, participants learned to explore the opposite of their initial beliefs by turning them around to possible opposites. For example, the initial belief “I am *not* able to study sufficiently” may be turned around to the opposite “I am able to study sufficiently.” by omitting the word “not” included in the initial belief. Then, participants were asked to find genuine proof of how the opposite could also be true for them.

**TABLE 1:** IBSR instructions.

**Investigate each of your statements, using the following question**	**Format of answer**
**Q1: Is this thought true?**	yes vs. no
**Q2: Can you absolutely know that this thought is true?**	yes vs. no
**Q3: How do you react, what happens when you have this thought?**	open
Does that thought bring peace or stress to your life?	open
What images do you see, past or future, as you think this thought?	open
What physical sensations arise having these thoughts and seeing these pictures?	open
What emotions arise when you have that thought?	open
Do any obsessions or addictions begin to appear when you have this thought (e.g., alcohol, drugs, shopping, food, and television)?	open
How do you treat others when you have this thought? How do you treat yourself when you have this thought?	open
**Q4: Who would you be without the thought?**	open
**Turn the thought around.** Example: My lecturer did not prepare me well enough for the exam.	
**Possible turnarounds:**	
**(1) To the self.** Example: I did not prepare me well enough for the exam.	open
**(2) To the other.** Example: I did not prepare my lecturer well enough for the exam.	open
**(3) To the opposite.** Example: My lecturer did prepare me well enough for the exam.	open
**Then find at least three specific, genuine examples of how each turnaround is true for you in the situation.**	open

After the respective seminars, participants of both groups received a diary. While participants of the *control group* were asked to further identify stressful situations and respective cognitions on a daily basis for 7 days, *intervention participants* were asked to explore their worry thoughts with the IBSR method for the same time interval. After the 7 days (i.e., approximately 1 week after the seminars; time 2) as well as immediately before the exams (time 3), dependent variables were measured again. After the exams, participants of the control group also received the IBSR intervention. All participants were debriefed and received additional information and materials regarding IBSR.

### Measures

To test if participants had chosen an exam that was actually important to them, we assessed the most frightening exams’ value with one item (“How important is this exam for you?”). Ratings were made using a 10-points scale ranging from 1 (*not at all important*) to 10 (*extremely important*).

Academic self-efficacy was assessed with a slightly modified version of the German Scale for the Assessment of Study Specific Self-Efficacy (Jerusalem and Schwarzer, 1986) using seven items (e.g., “Even though a test might be difficult, I know that I will pass it”). According to Bandura (1977), self-efficacy should be measured with a scale indicative of the academic behaviors necessary to accomplish the specific task at hand (i.e., passing a specific exam). The scale was thus modified to address students’ self-efficacy beliefs regarding a specific exam (e.g., “Even though *the* test might be difficult, I know that I will pass it“). All statements were rated using a 4-point scale from 1 (*absolutely not correct*) to 4 (*absolutely correct*). A mean self-efficacy score was calculated with high scores indicating high levels of self-efficacy. The items showed satisfactory internal consistencies (Cronbach’s α time 1 = 0.81, time 2 = 0.79, time 3 = 0.77).

Test anxiety was assessed with the German short version of the state scale of the State-Trait Anxiety Inventory (STAI-SKD; Bertrams and Englert, 2013). The STAI-SKD allows for the assessment of state test anxiety with five items (e.g., “I am tense”). Ratings were made using four-point scales from 1 (*not at all*) to 4 (*very much*). We used a mean score including all five items, with high scores indicating a high level of test anxiety. The items showed good internal consistencies (Cronbach’s α time 1 = 0.84, time 2 = 0.86, time 3 = 0.89).

Academic procrastination was measured with the German version of the Academic Procrastination State Inventory (APSI-d; Patzelt and Opitz, 2014). With its 23 items, the APSI-d asks how often certain procrastination thoughts and behaviors occurred during the previous week (e.g., “I have stopped learning prematurely to do something more pleasurable”). Participants rated these statements using a five-point scale from 0 (*never*) to 4 (*always*). A mean procrastination score was calculated with high scores indicating a high level of academic procrastination. The items showed excellent internal consistencies (Cronbach’s α time 1 = 0.90, time 2 = 0.93, time 3 = 0.93).

### Attrition Rate and Missing Data

Seventy-one participants completed baseline measures and attended the training modules (*n* IBSR = 40 vs. *n* control = 31). At the post-intervention measure, data of 66 participants (*n* IBSR = 38 vs. *n* control = 28) was assessed, while at the follow-up measure data of 57 participants was attained (*n* IBSR = 33 vs. *n* control = 24). Overall, there was an attrition rate of 19.7%. To test if the dropout was systematic, we created a dummy variable (code 1 = dropout, 0 = no dropout). A multivariate analysis of variance (MANOVA) with exam’s personal value, initial self-efficacy, initial test anxiety, and initial procrastination as dependent variables revealed a statistically non-significant overall multivariate effect of the dummy variable, *F*(4, 66) = 0.77, *p* = 0.550, η^2^_*partial*_ = 0.04. Also, there were no statistically significant univariate effects of the dummy variable, all *p*s > 0.160. A statistically insignificant χ^2^-test further showed, that dropout rates did not systematically differ between intervention group and control group, χ^2^(1) = 0.28, *p* = 0.593. These results indicate that the dropout was non-systematic.

Allover, 17.25% of data values were missing. Missing data ranged from a low of 1.2% to a high of 32.1% (e.g., for items assessing test anxiety at follow-up). To analyze the pattern of missing data, we calculated Little’s (1988) MCAR test, which resulted in a χ^2^(635) = 434.31, *p* = 0.999, indicating that data values were missing completely at random. In the following analysis, missing data was handled with the Full Information Maximum Likelihood Imputation (FIML) provided by Mplus (Muthén and Muthén, 1998-2012) for two reasons. First, the FIML procedure is preferable to listwise or pairwise deletion of missing data, which generally create biased parameter estimates as well as biased significance testing (Schlomer et al., 2010). Second, using the FIML procedure allows to retain the maximum amount of possible statistical power despite missing data.

### Data Analyses

**FIGURE 1:**
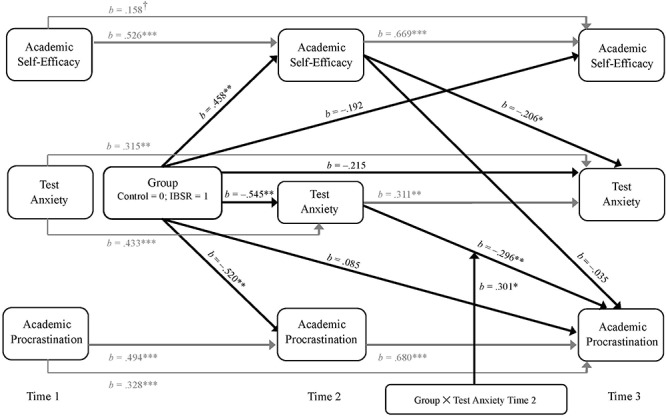
Path model of academic self-efficacy, test anxiety, and academic procrastination for all times of measurement. Depicted in gray color are first- and second-order autoregressive paths. Depicted in black color are direct effects of the IBSR intervention (dummy coded IBSR = 1 vs. control group = 0) on the dependent variables at time 2 and time 3, causal paths from academic self-efficacy measured at time 2 on test anxiety and academic procrastination measured at time 3, and from test anxiety measured at time 2 on academic procrastination measured at time 3. For increased readability, correlations between (residuals of) dependent variables were omitted in the graphical presentation of the model. Model fit: χ^2^(19) = 19.817, *p* = 0.406; CFI = 0.997; RMSEA = 0.025; SRMR = 0.081. All continuous variables were *z*-standardized. All reported parameter estimates are unstandardized. *N* = 71. ^†^*p* ≤ 0.10, ^∗^*p* ≤ 0.05, ^∗∗^*p* ≤ 0.01, ^∗∗∗^*p* ≤ 0.001. Reported are significance levels based one-tailed *p*-values.

Based on the theoretical assumptions of control-value theory (Pekrun, 2006) and TMT (Steel, 2007), we expected participants of the intervention group to report less test anxiety (H_2_) and less procrastination (H_3_) due to specific causal mechanisms (i.e., increased self-efficacy; H_1_). However, instead of investigating separate mediation models, we chose to use a path analysis including all variables and mediation paths (see Figure 1) due to the following reasons. Firstly, there is evidence that structural equation models perform better than simple regression models when it comes to investigate causal mechanisms via mediation analyses (Iacobucci et al., 2007). Secondly, the path analysis used in the present study allowed us to embed the focal mediation models into a longitudinal and nomological perspective. As a consequence, the path analysis was conducted with the software Mplus (Muthén and Muthén, 1998-2012). For the analysis, we applied the ML-estimator. When investigating the model fit, we relied on the guidelines given by Schermelleh-Engel et al. (2003) (acceptable model fit: RMSEA ≤ 0.08, CFI ≥ 0.95, SRMR ≤ 0.10; good model fit RMSEA ≤ 0.05, CFI ≥ 0.97, SRMR ≤ 0.05).

To account for the longitudinal design, we first included respective first order autoregressive paths for all three dependent variables and additionally second order autoregressive paths for the dependent variables between measures at time 1 and time 3 as suggested by Geiser (2013) and Newson (2015). Regarding time 1 and in accordance with previous empirical studies (e.g., Van Eerde, 2003; Steel, 2007) we further assumed positive correlations between test anxiety and procrastination as well as negative correlations between test anxiety/procrastination and self-efficacy. Additionally, we included correlations between residuals for time 2 and time 3 to account for individuals’ tendency to evaluate themselves as less effective (in terms of less self-efficacy and higher procrastination) when reporting higher test anxiety (and vice versa) at the same time of measurement (see Geiser, 2013).

To test for the experimental effects of the IBSR intervention, we coded a dummy variable (d) for which the control group was selected as the reference group (coded 0), while the IBSR group (coded 1) was contrasted with this reference group. In a first step, to investigate the immediate direct effects of IBSR on self-efficacy, test anxiety, and procrastination, we allowed for paths from the dummy variable (d) on all dependent variables measured at time 2. Secondly, to test the predicted causal interplay of self-efficacy, test anxiety, and procrastination between time 2 and time 3, we used a half longitudinal mediation design (Kline, 2016). To account for the *direct* effects of IBSR on all dependent variables immediately before the exams, we allowed for paths from the dummy variable d on all dependent variables measured at time 3. To investigate the expected *indirect* effects of IBSR on test anxiety (H_2_) before the exams via an increase in self-efficacy (H_1_), we included a path between self-efficacy measured at time 2 and test anxiety measured at time 3. To test for the expected *indirect* effects of IBSR on procrastination (H_3_) before the exams via an increase in self-efficacy and a decrease in test anxiety, we further included respective paths between self-efficacy/test anxiety measured at time 2 and procrastination measured at time 3.

Additionally, as we expected test anxiety to decrease for IBSR participants (due to the intervention) but to increase for control participants (due to approaching exams) at the last stages of the academic term (H_4_), we further included the two-way interaction of Group × Test Anxiety (measured at time 2) as a moderating variable for the path between test anxiety (time 2) and procrastination measured at time 3. To avoid the problems associated with multicollinearity between the predictor variable (i.e., Group), the moderator variable (i.e., Test Anxiety measured at time 2) and the respective interaction term (i.e., Group × Test Anxiety), all continuous variables were *z*-standardized as suggested by Frazier et al. (2004).

## Results

### Descriptive Statistics

Participants indicated the personal value of their most frightening exam to be very high (*M* = 8.15, *SD* = 1.52;

*M*_*intervention*_ = 8.21, *SD* = 1.27; *M*_*control*_ = 8.06, *SD* = 1.81). The respective frequency distribution was negatively skewed (–1.41, *SE* 0.029). Before the intervention, participants reported test anxiety of *M* = 2.80 (*SD* = 0.67), self-efficacy of *M* = 2.49 (*SD* = 0.56), and procrastination of *M* = 2.95 (*SD* = 0.64). Corresponding to the quasi-randomization, a multivariate analysis of variance with the factor Group (IBSR vs. control group) as independent variable revealed a non-significant overall multivariate effect, *F*(4, 66) = 0.87, *p* = 0.490, η^2^_*partial*_ = 0.05 on exam’s value, self-efficacy, test anxiety, and procrastination as dependent variables. Further, there were only non-significant univariate effects (all *p*s > 0.184), indicating that conditions did not differ regarding baseline levels of the analyzed variables. Zero-order correlations for the variables used in the path analyses are depicted in Table 2. As expected and in line with previous studies (Van Eerde, 2003; Steel, 2007), at all three times of measurement, these correlations suggest negative associations between self-efficacy and test anxiety, positive associations between procrastination and test anxiety, as well as negative relationships between self-efficacy and procrastination. Descriptive statistics for the dependent variables are reported separately for conditions and all points of measurement in Table 3.

**TABLE 2:** Zero-order correlations of dependent variables.

	**(1)**	**(2)**	**(3)**	**(4)**	**(5)**	**(6)**	**(7)**	**(8)**
**Pre-intervention (Time 1)**								
(1) Academic self-efficacy								
(2) Test anxiety	–0.34							
(3) Academic procrastination	–0.43	0.45						
**Post-Intervention (Time 2)**								
(4) Academic self-efficacy	0.57	–0.14	–0.41					
(5) Test anxiety	–0.27	0.46	0.35	–0.45				
(6) Academic procrastination	–0.30	0.31	0.60	–0.48	0.62			
**Follow-up (Time 3)**								
(7) Academic Self-Efficacy	0.58	–0.11	–0.45	0.74	–0.36	–0.44		
(8) Test Anxiety	–0.36	0.46	0.39	–0.41	0.59	0.50	–0.52	
(9) Academic Procrastination	–0.39	0.27	0.73	–0.42	0.39	0.80	–0.55	0.51

**N* = 71. Missing values were handled with FIML as provided by Mplus (Muthén and Muthén, 1998-2012).*

**TABLE 3:** Means and standard deviations for academic self-efficacy, test anxiety, and academic procrastination for IBSR intervention and control group.

	**Pre-intervention (*n* = 71)**		**Post-intervention (*n* = 66)**		**Follow-up (*n* = 57)**	
	***M***	***SD***	***M***	***SD***	***M***	***SD***
**Academic self-efficacy**						
IBSR Intervention	2.45	0.60	2.75	0.54	2.65	0.55
Control	2.53	0.52	2.55	0.47	2.53	0.51
Overall	2.49	0.56	2.66	0.52	2.60	0.53
**Test anxiety**						
IBSR Intervention	2.80	0.74	2.30	0.58	2.45	0.65
Control	2.81	0.58	2.67	0.75	2.84	0.72
Overall	2.80	0.67	2.46	0.68	2.62	0.70
**Academic procrastination**						
IBSR Intervention	2.87	0.70	2.46	0.66	2.60	0.67
Control	3.07	0.54	2.91	0.67	2.90	0.61
Overall	2.95	0.64	2.66	0.70	2.72	0.66

### Preliminary Data Analyses

We had expected the IBSR intervention to increase participants’ self-efficacy, to reduce participants’ test anxiety as well as their procrastination in comparison to the control group. Thus, as preliminary analyses, we conducted three separate analyses of covariance with Group (IBSR vs. control group) as between-subjects factor and with self-efficacy, test anxiety, and procrastination measured after the intervention as respective dependent variables. As recommended by Van Breukelen (2006), we also included the baseline values of each respective dependent variable as a covariate. Results revealed a statistically significant effect of the IBSR intervention on participants’ self-efficacy, *F*(1/63) = 5.49, *p* = 0.022, η^2^_*partial*_ = 0.08, on participants’ test anxiety *F*(1/63) = 6.56, *p* = 0.013, η^2^_*partial*_ = 0.09, and their procrastination *F*(1/62) = 5.85, *p* = 0.019, η^2^_*partial*_ = 0.09. These results provide first preliminary evidence for the expected effects of the IBSR intervention.

### Data Screening Procedure

As structural equation modeling procedures are susceptible to abnormalities in the data (Kline, 2016), we examined if the data met the necessary requirements. To identify potential outliers, we first inspected the frequency distribution of the *z*-scores for all variables used in the statistical analyses. Applying the rule of |*z*| > 3.29 (Tabachnick and Fidell, 2014), we did not detect any outliers in the data. Second, we relied on the variance inflation factor (VIF) to test for extreme collinearity. VIF values for all variables were lower than the threshold of 10.00 (all VIFs < 5.15). Therefore, extreme collinearity did not occur in the data. Third, to ensure the requirement of multivariate normality, we inspected the univariate frequency distributions for all variables (for all results see Table 4). Shapiro–Wilk tests were statistically non-significant for all variables except for test anxiety measured at time 2. However, visual inspection of the respective frequency distribution showed that it was close to normality. Based on the suggestion by Kline (2016) we proceeded to analyze that data using structural equation modeling without transformation of the respective variable.

**TABLE 4:** Univariate statistics for academic self-efficacy, test anxiety, and academic procrastination.

	**Pre-intervention**	**Post-intervention**	**Follow-up**
	
	***n* = 71**	***n* = 65**	***n* = 57**
**Academic Self-efficacy**			
Skew (SE)	0.104 (0.285)	–0.437 (0.297)	–0.035 (0.316)
Kurtosis (SE)	–0.400 (0.563)	0.129 (0.586)	–0.020 (0.623)
Significance Shapiro–Wilk Test	0.730	0.054	0.372
**Test Anxiety**			
Skew (SE)	–0.239 (0.285)	0.757 (0.297)	0.019 (316)
Kurtosis (SE)	–0.602 (0.563)	–0.243 (0.586)	–0.518 (0.623)
Significance Shapiro–Wilk Test	0.062	0.000	0.533
**Academic Procrastination**			
Skew (SE)	0.275 (0.285)	0.541 (0.297)	0.565 (0.316)
Kurtosis (SE)	0.174 (0.563)	–0.012 (0.586)	–0.013 (0.623)
Significance Shapiro–Wilk Test	0.738	0.199	0.159

### Direct and Indirect Effects of IBSR

The fit statistics of the model were acceptable to good, χ^2^(19) = 19.817, *p* = 0.406; CFI = 0.997; RMSEA = 0.025; SRMR = 0.081. Following the suggestion of Hayes (2013), we only report unstandardized coefficients for all paths as standardized coefficients are not meaningful due to the dichotomous character of the group variable d (IBSR vs. control group). In the following, we report one-tailed *p*-values. Results provide evidence for the stability of all three dependent variables over time. With the exception of the second order autoregressive path for self-efficacy time 3 (*b* = 0.158, *SE* = 0.118, *p* = 0.091), all other coefficients of first and second order autoregressive paths were statistically significant at a *p*-level of 0.05. As expected and in line with previous studies (Van Eerde, 2003; Steel, 2007), at all three times of measurement, we found self-efficacy and test anxiety to be negatively correlated (time 1: *b* = –0.334, *SE* = 0.123, *p* = 0.004; time 2: *b* = –0.213, *SE* = 0.062, *p* < 0.001; time 3: *b* = –0.208, *SE* = 0.074, *p* = 0.003), procrastination and test anxiety to be positively associated (time 1: *b* = 0.439, *SE* = 0.137, *p* < 0.001; time 2: *b* = 0.273, *SE* = 0.089, *p* = 0.001; time 3: *b* = 0.126, *SE* = 0.052, *p* = 0.008), and negative relationships between self-efficacy and procrastination (time 1: *b* = –0.428, *SE* = 0.136, *p* = 0.001; time 2: *b* = –0.172, *SE* = 0.065, *p* = 0.004; time 3: *b* = –0.122, *SE* = 0.046, *p* = 0.004).

#### Direct and Indirect Effects of IBSR on Self-Efficacy (H_1_)

Right after the intervention, in line with our prediction, IBSR participants reported statistically significantly enhanced self-efficacy (*a*_1_ = 0.458, *SE* = 0.190, *p* = 0.008) in comparison to the control group. At the end of the term, the IBSR intervention no longer directly affected self-efficacy (*c*_1_*’* = –0.192, *SE* = 0.173, *p* = 0.133). However, participants who had reported higher self-efficacy immediately after the intervention also reported higher self-efficacy at the end of the academic term (*b*_11_ = 0.669, *SE* = 0.120, *p* < 0.001). A bias-corrected 95% bootstrap confidence interval (BCI) for the indirect effect (*a*_1_*b*_11_ = 0.306) based on 10.000 bootstrap samples was entirely above zero (0.069 to 0.613). This indicates the IBSR intervention indirectly enhanced self-efficacy and that this effect lasted until the end of the term.

#### Direct and Indirect Effects of IBSR on Test Anxiety (H_2_)

Right after the intervention, IBSR participants reported statistically significantly less test anxiety (*a*_2_ = –0.545, *SE* = 0.221, *p* = 0.007) than participants of the control group. At the end of the term, the IBSR intervention no longer directly affected test anxiety (*c*_2_*’* = –0.215, *SE* = 0.225, *p* = 0.170). However, we found an indirect effect of the IBSR intervention. Firstly, participants who had reported less test anxiety after the intervention also reported less test anxiety at the end of the academic term (*b*_22_ = 0.311, *SE* = 0.121, *p* = 0.005). A bias-corrected 95% BCI for the indirect effect (*a*_2_*b*_22_ = –0.169) was entirely under zero (–0.453 to –0.030). Secondly and as expected, participants who reported more self-efficacy after the intervention also reported statistically significant less test anxiety later on (*b*_21_ = –0.206, *SE* = 0.118, *p* = 0.040). A bias-corrected 90% BCI for the indirect effect (*a*_1_*b*_21_ = –0.094) was entirely under zero (–0.252 to –0.013). The total indirect effect of IBSR on test anxiety was also statistically significant (*b*_2_ = –0.264, *SE* = 0.115, *p* = 0.011), with its 95% BCI completely under zero (–0.542 to –0.078). Thus, the IBSR intervention reduced test anxiety at the end of the academic term indirectly via an immediate increase in self-efficacy and an immediate decrease in test anxiety.

#### Direct and Indirect Effects of IBSR on Procrastination (H_3_)

Right after the intervention, IBSR participants reported statistically significantly less procrastination (*a*_3_ = –0.520, *SE* = 0.199, *p* = 0.005) than participants of the control group. Even though the IBSR intervention no longer directly affected procrastination (*c*_3_*’* = 0.085, *SE* = 0.146, *p* = 0.279) at the end of the term, we found an indirect effect of the IBSR intervention on procrastination, which is – as predicted – more complex in its nature. Firstly, the initial reduction of procrastination resulted in a lasting reduction of procrastination as participants who had reported less procrastination after the intervention also reported less procrastination at the end of the term (*b*_33_ = 0.680, *SE* = 0.107, *p* < 0.001). A bias-corrected 95% BCI for this specific indirect effect of IBSR (*a*_3_*b*_33_ = –0.354) was entirely under zero (–0.682 to –0.104). Secondly, and in contrast to our hypotheses, results revealed that participants with increased self-efficacy did not report statistically significant less procrastination at the end of the academic term (*b*_31_ = –0.035, *SE* = 0.088, *p* = 0.348). Accordingly, the specific indirect effect (*a*_1_*b*_31_ = –0.016) was statistically non-significant as confirmed by the bias-corrected 90% BCI (–0.107 to 0.040).

**FIGURE 2:**
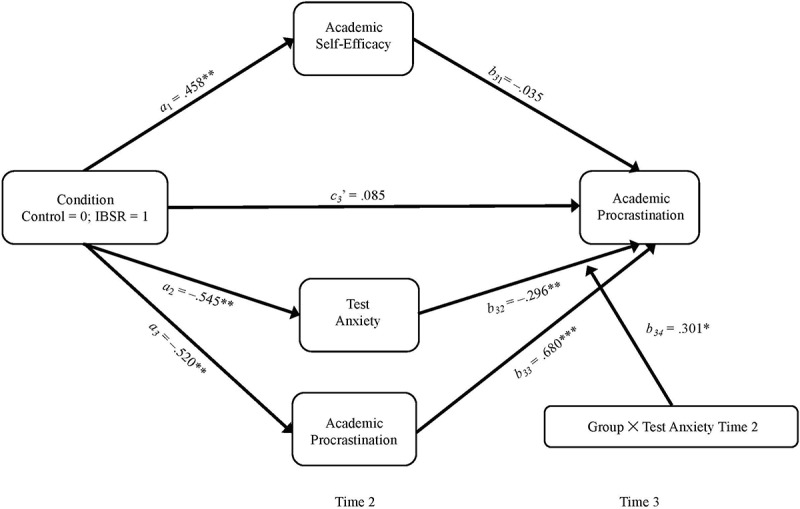
Conditional Parallel Multiple Mediation Models for Academic Procrastination Measured at Time 3. All continuous variables were *z*-standardized. All reported parameter estimates are unstandardized. *N* = 71. ^†^*p* ≤ 0.10, ^∗^*p* ≤ 0.05, ^∗∗^*p* ≤ 0.01, ^∗∗∗^*p* ≤ 0.001. Reported are significance levels based one-tailed *p*-values.

Thirdly, we predicted IBSR to indirectly reduce procrastination via an immediate *reduction* of test anxiety for participants of the intervention. Also, we had predicted that participants of the control group should demonstrate an *increase* of test anxiety and this increase in test anxiety to reduce procrastination (H_4_). Thus, a significant coefficient was expected for the two-way interaction of Group × Test Anxiety (time 2). Results revealed that participants who had reported *more* test anxiety at time 2, reported statistically significant *less* procrastination at the end of the academic term (*b*_32_ = –0.296, *SE* = 0.098, *p* = 0.002). This effect was qualified by the predicted effect of the interaction of Group × Test Anxiety (*b*_34_ = 0.301, *SE* = 0.156, *p* = 0.027) which shows that experimental conditions had a differential effect on the causal relationship between test anxiety measured at time 2 and procrastination measured at time 3 (see Figure 2). For the control group, simple slopes analyses revealed a statistically significant effect of test anxiety (time 2) on procrastination (time 3) [*b*_34__(__0__)_ = –0.296, *SE* = 0.098, *p* = 0.002, 95% BCI (–0.491 to –0.103)], while there was no effect for the IBSR group [*b*_34__(__1__)_ = 0.005, *SE* = 0.145, *p* = 0.487, 90% BCI (–0.233 to 0.232)]. Altogether, for the IBSR group, we found a statistically significant *total* indirect effect of IBSR on procrastination [*b*_3__(__1__)_ = –0.372, *SE* = 0.169, *p* = 0.014, 95% BCI (–0.751 to –0.083)], but not for the control group [*b*_3__(__0__)_ = –0.208, *SE* = 0.130, *p* = 0.055, 90% BCI (–0.423 to 0.005)]. Thus, the IBSR intervention reduced procrastination at the end of the academic term indirectly, mainly via an immediate reduction of procrastination. For all results of the mediation analyses see Table 5.

**TABLE 5:** Regression coefficients, standard errors, and model summary information for the conditional parallel multiple mediation models for academic procrastination measured at time 3.

		**Consequent**										
		***M*_1_ (Self-efficacy time 2)**				***M*_2_ (Test anxiety time 2)**				***M*_3_ (Procrastination time 2)**		
**Antecedent**		**Coeff.**	***SE***	***p***		**Coeff.**	***SE***	***p***		**Coeff.**	***SE***	***p***
*X* (IBSR vs. Control)	*a*_1_	0.458	0.190	0.008	*a*_2_	–0.545	0.221	0.007	*a*_3_	–0.520	0.199	0.005
Mediator Value Time 1		0.526	0.092	0.000		0.433	0.090	0.000		0.494	0.093	0.000
Constant	*i*_*M*__1_	–0.260	0.137	0.029	*i*_*M*__2_	0.308	0.187	0.050	*i*_*M*__3_	0.280	0.154	0.035
	*R*^2^ = 0.338					*R*^2^ = 0.294				*R*^2^ = 0.373		

		**Consequent**										
		***Y*_1_ (Self-Efficacy Time 3)**				***Y*_2_ (Test Anxiety Time 2)**				***Y*_3_ (Procrastination Time 2)**		
**Antecedent**		**Coeff.**	***SE***	***p***		**Coeff.**	***SE***	***p***		**Coeff.**	***SE***	***p***

*X* (IBSR vs. Control)	*c*_1_*’*	–0.192	0.173	0.133	*c*_2_*’*	–0.215	0.225	0.170	*c*_3_*’*	0.085	0.146	0.279
Mediator Value Time 1		0.158	0.118	0.091		0.315	0.111	0.002		0.328	0.087	0.000
*M*_1_ (Self-Efficacy Time 2)	*b*_11_	0.669	0.120	0.000	*b*_21_	–0.206	0.118	0.040	*b*_31_	–0.035	0.088	0.348
*M*_2_ (Test Anxiety Time 2)					*b*_22_	0.311	0.121	0.005	*b*_32_	–0.296	0.098	0.002
*M*_3_ (Procrastination Time 2)									*b*_33_	0.680	0.107	0.000
*X* × *M*_2_									*b*_34_	0.301	0.156	0.027
Constant	*i*_1_	0.124	0.129	0.168	*i*_2_	0.115	0.185	0.266	*i*_3_	0.018	0.099	0.428
	*R*^2^ = 0.559					*R*^2^ = 0.421				*R*^2^ = 0.754		

**N* = 71. Experimental condition was dummy-coded (IBSR group = 1 vs. control group = 0). All continuous variables were *z*-standardized. Reported are unstandardized coefficients. Reported significance levels are based one-tailed *p*-values.*

## Discussion

In the present research, we investigated the effects of an IBSR short intervention on test anxiety, procrastination, and self-efficacy as well as their causal interplay in the last part of an academic term in a sample of university students suffering from test anxiety and procrastination. We had predicted the IBSR intervention to enhance self-efficacy (H_1_) and this increase in self-efficacy – subsequently – to reduce test anxiety (H_2_) and procrastination (H_3_) for participants of the IBSR intervention. In contrast, we had also expected a decrease in procrastination for participants of the control group, but this decrease rather to be a consequence of an increase in test anxiety due to the approaching exams and deadlines (H_4_). Results of the data analyses mostly support our hypotheses. Firstly, in accordance with our first hypothesis, results showed that the IBSR intervention increased participants’ self-efficacy and this effect to be stable until the end of the academic term. We interpret these results in accordance with the theoretical assumptions of CEST (Epstein, 2003) and self-efficacy theory (Bandura, 1977). Based on the rationale of both theories, we assume that information processing leading to cognitive appraisals (such as self-efficacy beliefs) is not only informed by conscious and rational reasons (i.e., verbal persuasion), but also by experience-based information stemming from experiential schemas and knowledge such as (vicarious) mastery experiences and current experience of physical arousal. Following from this, a permanent modification of cognitive appraisals (such as low self-efficacy beliefs) is assumed to follow from rational debate as well as from new – efficacy-enhancing – experiences. As IBSR allows for a debate of cognitive appraisals such as low self-efficacy beliefs through experiential self-exploration (e.g., by imagination of the testing situation without the distortions caused by participants’ low self-efficacy beliefs) and rational persuasion (e.g., through exploration of the validity of high self-efficacy beliefs), individuals should show increased self-efficacy after participation in an IBSR intervention. Secondly, we found a stable decrease in test anxiety for participants of the IBSR intervention, which was partly due to increased self-efficacy. This result is in line with control-value theory (Pekrun, 2006), which states that test anxiety is caused by cognitive appraisals including (low) self-efficacy beliefs and matches existing empirical evidence showing the potential of IBSR to reduce test anxiety (Krispenz and Dickhäuser, 2018). Thirdly, and for all participants, we found a lasting decrease in academic procrastination. However, in line with our assumptions, data analyses revealed this decrease in procrastination to be caused by different mechanisms for the respective groups. For the control group and as predicted by our fourth hypothesis, the reduction of procrastination was due to an *increase* in test anxiety [*b*_34__(__0__)_ = –0.296, *SE* = 0.098, *p* = 0.002]. We interpret this effect in line with studies which found students to experience higher levels of anxiety (Lay et al., 1989) before exams when they had delayed studying earlier in the semester and in accordance with TMT (Steel, 2007), which assumes students to procrastinate less in the last stages of an academic term – as deadlines approach, students are forced to study for their exams if they want to avoid failing due to poor preparation. For intervention participants, results also revealed a long-term decrease in procrastination. However, this effect was – contrary to our third hypothesis – neither caused by an increase in self-efficacy nor by a (subsequent) decrease in test anxiety. Rather, the decrease in procrastination was caused by an immediate effect of the IBSR intervention on procrastination. In particular, we found the relationship between test anxiety and procrastination to completely vanish for IBSR participants in the time between the intervention till the end of the academic term [*b*_34__(__1__)_ = 0.005, *SE* = 0.145, *p* = 0.487]. From this, we conclude that the IBSR method might have provided participants of the IBSR intervention with new means for emotional coping: As test anxiety is accompanied by states of unpleasant physical arousal and worry thoughts (Pekrun, 2006), students often feel the desire to withdraw from the anxiety-causing situation (Geen, 1987; Matthews et al., 1999). However, during the IBSR intervention seminars, test anxious students were taught to investigate their worry thoughts and to explore any accompanying unpleasant feelings and sensations with the IBSR method (Question 3; see Table 1). Additionally, students were enabled to mentally experience the anxiety-causing situation without the distortions caused by their worry thoughts (Question 4; see Table 1), which should allow them a new and potentially more positive experience (e.g., feelings of relief or curiosity). Therefore, when confronted with the unpleasant state of test anxiety after the IBSR intervention, IBSR participants might have no longer felt the need to withdraw from the situation through procrastination. Rather, they might have applied the IBSR method as an alternative coping strategy to deal with unpleasant physical arousal and worry thoughts. Nevertheless, additional data is needed to confirm this assumption.

The results of our study significantly contribute to the literature on IBSR. For the first time, they show that IBSR is potent not only in reducing test anxiety (Krispenz and Dickhäuser, 2018), but also in enhancing academic self-efficacy and reducing academic procrastination. Our research also demonstrates that these effects last longer than 2 days and remain stable especially in the last stage of an academic term. The present study also overcomes the limitations of previous studies on IBSR. On the one hand and in contrast to Krispenz and Dickhäuser (2018), we assisted IBSR participants in their first practice of IBSR, thereby avoiding any difficulties participants unfamiliar with IBSR might encounter when applying the IBSR method for the first time. On the other hand, in previous studies (e.g., Smernoff et al., 2015), participants usually attended a 9-day IBSR intervention making participation extremely time-consuming. In our study, the IBSR intervention lasted only 3 h which shows that participants can be trained to use the IBSR method more effectively.

### Limitations

There are limitations to the present study which need to be acknowledged. Firstly, and even though we found IBSR to decrease procrastination, we did not find this decrease to be caused by the found increase in self-efficacy as TMT (Steel, 2007) would predict. This result might be due to the fact that participants were not explicitly instructed to investigate only (low) self-efficacy beliefs but anxiety causing worry thoughts *per se*. Hence, future studies interested in further investigating the positive effects of IBSR on procrastination via an enhancement in self-efficacy could profit from guiding participants in identifying and exploring self-efficacy beliefs only. Also, at first view, the causal interpretation of the found effects is limited due to the fact that the present study did use a quasi-randomized control trial. Due to practical reasons, participants were assigned to the conditions (IBSR vs. control group) by choosing from the four possible intervention dates without knowing on what dates the IBSR intervention was actually scheduled. However, there is no reason to assume that participants preferences for dates was systematically associated with one of the outcome variables. This is also confirmed by the results showing that experimental groups did not statistically differ in their initial levels of the dependent variables. Further, the design of the present study allows for a rather conservative estimation of the effects of IBSR. We included an active control group instead of a neutral inactive control group. In particular, participants in the control group were completing activities (i.e., a 3-h seminar and a diary) which could have helped them to increase their self-efficacy and reduce their test anxiety and procrastination long-term. This might explain why some of the long-term direct effects of the IBSR intervention were not statistically significant when compared to the control group. To exclude this alternative explanation of the present results, future studies should use a 3-group design including an intervention group, an active control group, and a neutral control group. Also, instead of a general student population (see Krispenz and Dickhäuser, 2018), the present study explicitly addressed university students suffering from test anxiety and/or academic procrastination. Nevertheless, the IBSR intervention was given to all students who were interested in participating regardless of their initial levels of test anxiety. Future studies should investigate if the effects found in the present study are replicable in a sample of highly test anxious students.

Another important limitation of the present study is that we used complex path analysis to investigate the relationships among the variables of interest by using a sample of *N* = 71. As a consequence of the sample size, we were not able to specify latent variables in the model. Thus, future research should replicate the results of the present study by repeating the procedure with a larger sample to allow for analyzes of both observed and latent variables. Furthermore, the measurement of the dependent variables was restricted to the second part of an academic term. Accordingly, future studies should on the one hand investigate the causal interplay between self-efficacy, test anxiety, and procrastination over the course of a whole academic term. On the other hand, they should include an even longer follow-up period to investigate if the found effects hold even over a longer time period. Finally, future research should include additional measures related to self-efficacy, test anxiety, and procrastination such as academic performance (e.g., grades).

## Conclusion

The present study provides preliminary evidence that IBSR is potent in enhancing self-efficacy as well as in reducing test anxiety and procrastination in a sample of university students suffering from test anxiety and procrastination. These findings have important practical implications for educational settings as students suffering from both phenomena might easily profit from learning the IBSR method considering that the method is guided by a simple and clear defined set of questions, allowing for a structured way of *self*-inquiry. As a consequence, the practice of IBSR does not require a therapeutic setting. This makes the IBSR method easily available and potentially helpful to anyone who wants to change their negative thinking.

## Data Availability

The datasets generated for this study are available on request to the corresponding author.

## Ethics Statment

Ethical review and approval was not required for the study on human participants in accordance with the local legislation and institutional requirements. The patients/participants provided their written informed consent to participate in this study.

## Author Contributions

AK, CG, and LS developed the study concept and design, and collected the data. AK and OD analyzed and interpreted the data. AK drafted the manuscript. OD, CG, and LS critically revised the manuscript. All authors approved the final version to be published, and agreed to be accountable for all aspects of the work in ensuring that questions related to the accuracy or integrity of any part of the work are appropriately investigated and resolved.

## Conflict of Interest Statement

The authors declare that the research was conducted in the absence of any commercial or financial relationships that could be construed as a potential conflict of interest.

## Footnotes

1 Note that test anxiety and self-efficacy were also assessed immediately after the initial intervention. Since there was no matching score for procrastination due to the specifics of the APSI-d (Patzelt and Opitz, 2014) those data were not included in the data analyses. Further, since the present study was part of a bigger research project, additional data was collected measuring moderating variables such as self-compassion, mindfulness, and the big five personality traits. The results of the respective data will be published in another paper and were also not included in the present data analyses.
